# What is the role of the hippocampus and parahippocampal gyrus in the persistence of tinnitus?

**DOI:** 10.1002/hbm.26627

**Published:** 2024-02-20

**Authors:** Joel I. Berger, Alexander J. Billig, William Sedley, Sukhbinder Kumar, Timothy D. Griffiths, Phillip E. Gander

**Affiliations:** ^1^ Department of Neurosurgery University of Iowa Iowa City Iowa USA; ^2^ Ear Institute University College London London UK; ^3^ Biosciences Institute Newcastle University Newcastle UK; ^4^ Department of Radiology University of Iowa Iowa City Iowa USA

**Keywords:** auditory, hippocampal, memory, neuroscience

## Abstract

The hippocampus and parahippocampal gyrus have been implicated as part of a tinnitus network by a number of studies. These structures are usually considered in the context of a “limbic system,” a concept typically invoked to explain the emotional response to tinnitus. Despite this common framing, it is not apparent from current literature that this is necessarily the main functional role of these structures in persistent tinnitus. Here, we highlight a different role that encompasses their most commonly implicated functional position within the brain—that is, as a memory system. We consider tinnitus as an auditory object that is held in memory, which may be made persistent by associated activity from the hippocampus and parahippocampal gyrus. Evidence from animal and human studies implicating these structures in tinnitus is reviewed and used as an anchor for this hypothesis. We highlight the potential for the hippocampus/parahippocampal gyrus to facilitate maintenance of the memory of the tinnitus percept via communication with auditory cortex, rather than (or in addition to) mediating emotional responses to this percept.

## Graphical abstract

The proposed model by which hippocampal activity may be preserved when hearing loss is present with tinnitus. In hearing loss, degraded communication between auditory cortex and medial temporal lobe regions may occur (top panel). In tinnitus, communication may be somewhat preserved, due to a perpetual updating of the tinnitus signal (bottom panel).

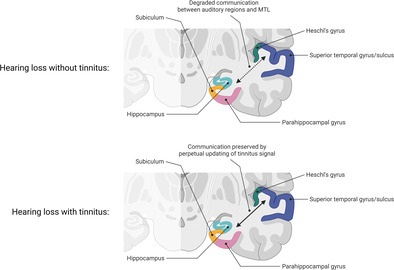

## 1 INTRODUCTION

Subjective tinnitus, a widespread societal and health issue, is commonly defined as the perception of sound in the absence of an external acoustic stimulus. In one of the most popular models of tinnitus (Jastreboff, 1990), focus was drawn to the role of limbic structures (specifically hippocampus, parahippocampal gyrus [PHG], and amygdala) in tinnitus. In that model, and in subsequent papers, the emphasis has often been around the emotional functions of these structures, wherein their role is implicated in the negative emotional reinforcement of tinnitus (see Georgiewa et al., 2006 and Mazurek et al., 2019 for relevant reviews). Some of these suggestions are supported by tinnitus subjects showing elevated parahippocampal activity in response to certain affective stimuli (although interestingly not in the amygdala; Carpenter‐Thompson et al., 2014; Davies et al., 2017). However, in other fields, the hippocampus and PHG are most commonly highlighted as involved in the storage and retrieval of long‐term memory (Squire & Zola‐Morgan, 1991), spatial memory (O'Keefe et al., 1998), and an increasing number of cognitive functions (e.g. Ruiz et al., 2020). Furthermore, as some authors pointed out over two decades ago, the term “limbic system” is poorly specified and can create scientific misdirection (Kotter & Stephan, 1997), particularly when using the label “limbic region/system” to refer generally to affective processing, rather than a specific known function of the involved brain regions. Indeed, the term “limbic system” was originally coined by Paul MacLean as another term for the visceral brain (MacLean, 1949; Maclean, 1952), which naturally creates a focus on the involvement of these structures in underlying emotional responsivity. It should be noted that other tinnitus theories have implicated these structures in a way that does not focus on an emotional aspect, but instead creates greater susceptibility to tinnitus (i.e., vulnerability) when damage is present in the limbic system in conjunction with hearing loss (Rauschecker et al., 2015).

**FIGURE 1:**
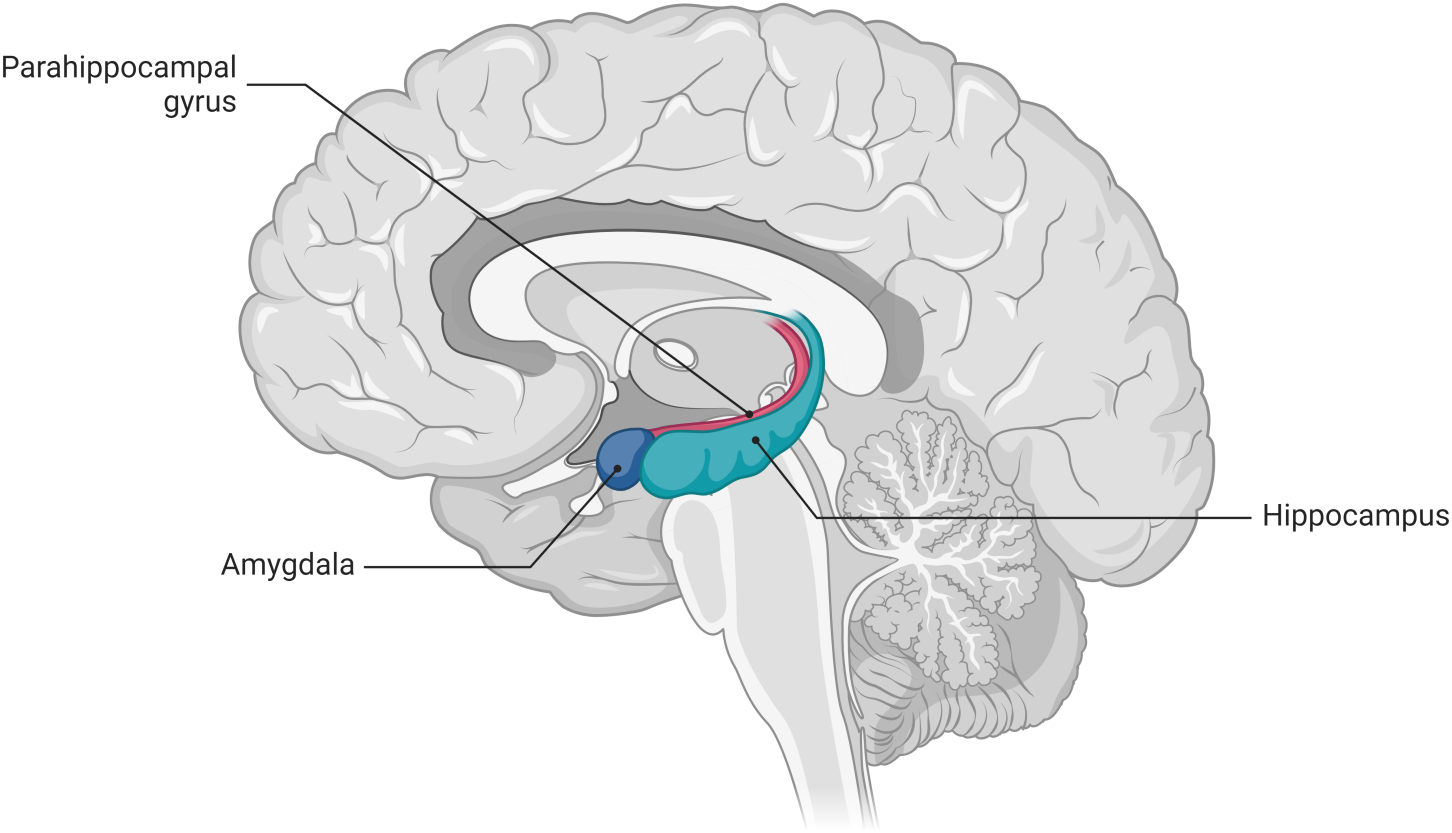
Key structures in the medial temporal lobe.

Here, we examine literature implicating the hippocampal and parahippocampal regions in the persistence of tinnitus, and discuss the mechanistic role that these structures may play in the perception of chronic tinnitus, with the focus related to these structures forming part of a memory network (Squire et al., 2004). The hippocampus and PHG are within the medial temporal lobe (MTL), a collection of cortical and subcortical structures adjacent the mesial aspect of the temporal lobe (Oler, 2011; Figure 1). The MTL consists of the hippocampal formation (CA1—CA4, dentate gyrus, and subiculum), PHG (perirhinal, entorhinal, and parahippocampal cortices), and amygdala, in each cerebral hemisphere, with one of the main associated functions being that of a memory system (Squire et al., 2004).

The anatomical connections between auditory cortex and MTL, including the hippocampus and PHG, have been reviewed elsewhere (Billig et al., 2022; Kraus & Canlon, 2012), along with how noise exposure (Kraus & Canlon, 2012) and plasticity within these connections (Kapolowicz & Thompson, 2019) may relate to tinnitus. Here, we posit a model of the hippocampus and PHG in mnemonic processing of the tinnitus percept. Notably, while models of tinnitus involving the MTL have been put forward previously by Shulman (1995), and expanded upon later in Shulman et al. (2009) and de Ridder et al. (2011), these often intertwine memory processes with emotional processing (with the exception of Salvi et al., 2011)—of relevant importance to this, an intact MTL is not required to experience rich emotional processing (Damasio et al., 2013). We examine this model with evidence from various literature, particularly including data acquired over the last decade in both animals and humans, in an attempt to better understand specifically how the hippocampus and PHG may contribute to tinnitus persistence (Hockley & Shore, 2023; Zhang et al., 2022).

For the purposes of this review, we define “persistence” as the mechanisms responsible for the constant or frequent presence of tinnitus over a long period of time, that is, “chronic” tinnitus. The ambiguity of this definition reflects the lack of an agreed or evidence‐based definition of chronic tinnitus. Arbitrarily, 3‐ or 6‐months' duration are typically used as definitions in tinnitus research (e.g. Wu et al., 2018), but there is evidence that tinnitus persistent for 4 weeks has >75–90% chance of persisting into this timeframe (Olderog et al., 2004; Vielsmeier et al., 2020; Wallhäusser‐Franke et al., 2017), and thus might already reasonably be considered “persistent.” Therefore, the main distinction from “persistent” tinnitus is “transient” tinnitus, occurring for seconds to days either spontaneously or following an insult to the auditory periphery.

### 1.1 The role of MTL in auditory object formation and memory

To consider the potential functional implications of changes in the MTL accompanying tinnitus, it is useful to consider its role in normal object formation. In humans and monkeys, perirhinal cortex is important in processing conjunctions of visual features both for perception and memory (Barense et al., 2007; Buckley et al., 2001; Bussey et al., 2002; Lee et al., 2005), while hippocampus is critical for establishing relationships between visual objects in a scene. In audition, MTL structures track the number of perceived objects based on simultaneous (Alain et al., 2001) or sequential (Curtu et al., 2019) grouping of sound elements, and damage to perirhinal cortex in rats affects their ability to bind discontinuous vocalisations into a whole (Bang & Brown, 2009). The hippocampus is also involved in bridging temporal gaps during auditory working memory (Kumar et al., 2016; Kumar et al., 2021) and trace conditioning (Clark & Squire, 1998; Solomon et al., 1986). Along with a role in forming auditory objects, the hippocampus can also convey predictions to auditory cortex of how such objects or events, such as spoken sentences, will unfold over time. As one example, in an intracranial study (Michelmann et al., 2021), patients were presented with a spoken story twice. Upon the second listening, predictive recall signals were evident between the hippocampus and auditory cortex, with the hippocampal activity preceding the cortical activity by an average of 740 ms. Anatomical and physiological evidence for the relevant pathways has been reviewed by Billig et al. (2022), along with the range of contributions made by hippocampus to normal sound processing.

### 1.2 Changes in the hippocampus following hearing loss

We begin by considering how the hippocampus would function in the presence of degraded auditory input alone, before focusing on a relationship to the conscious percept of tinnitus. This is important as even though changes in the hippocampus may occur in tinnitus, the high comorbidity of tinnitus with other conditions (summarised in Mazurek et al., 2023) means that these other conditions need to be considered as confounds when considering the literature. Given that there are likely changes relating to hearing loss alone, an important note is that many human tinnitus studies have not controlled for hearing loss, which is likely to be a significant confounding factor (Adjamian et al., 2014), and even when they do account for hearing loss, this is done with varying levels of control. Therefore, we state explicitly below when hearing loss is accounted for in a particular study, though it is important for future studies to explicitly consider and attempt to account for this confound, which may explain some of the heterogeneity in the literature. Indeed, there is in fact a relative scarcity of literature relating changes in the MTL purely directly to hearing loss without the presence of tinnitus, and even when examining the effects of hearing loss on the central nervous system this area is often overlooked as a region of interest (e.g. Giroud et al., 2018). For instance, while examination of resting‐state neural activity is a mainstay of tinnitus research, this is rarely used to study hearing loss alone.

Hippocampal neurogenesis has been found to be impaired in mouse models following noise exposure (Liu et al., 2016; Liu et al., 2018; Park et al., 2018) and conductive hearing loss (Kurioka et al., 2021), particularly in the case of bilateral conductive loss. In a rat model of chronic noise exposure, impaired hippocampal neurogenesis was evident (Cui et al., 2009), concurrent with increases in phosphorylated tau and lipofuscin (markers of neurofibrillary degeneration and age‐related lysosomal degradation, respectively), and related to a decline in behavioural cognitive abilities. In one study examining this using fMRI in humans, presbycusis (age‐related hearing loss) was found to be associated with decreased amplitudes of low‐frequency (0.01–0.08 Hz) fluctuation and regional homogeneity in right PHG during the resting state (Chen, Chen, et al., 2018), respectively, reflecting measures of low‐frequency blood‐oxygen‐level‐dependent (BOLD) activity and local neural synchrony. Another resting‐state fMRI study suggested that changes from presbycusis may be accounted for by the increased attentional listening effort demands placed on individuals with hearing loss, rather than intrinsic changes in functional connectivity caused by hearing loss (Rosemann & Thiel, 2019). Several other studies have shown that hippocampal volume is associated with peripheral hearing abilities and hippocampal/PHG atrophy is accelerated in hearing loss, even without confirmed presence of tinnitus (e.g. Uchida et al., 2018; Belkhiria et al., 2019; Xu et al., 2019; Shim et al., 2023; for a review, see Jafari et al., 2021). Thus, at a minimum, changes due to hearing loss alone must be identified in order to prevent these being misattributed to mechanisms of tinnitus. Furthermore, as the majority of cases of tinnitus follow hearing loss, such changes might also be contributory towards tinnitus, but insufficient without additional changes.

## 2 TINNITUS‐SPECIFIC CHANGES

Table 1 summarises the literature showing various changes in the hippocampus/PHG related to tinnitus, as discussed below. We will first highlight studies involving humans, showing structural changes (Section 2.1.) and functional changes (Section 2.2.), and then studies involving animals (Section 2.3.). Following this, we will outline a model integrating the current evidence for involvement of the hippocampus in the persistence of a tinnitus memory (Section 3).

**TABLE 1:** Summary of various studies included here that examined changes in the hippocampus/PHG related to tinnitus. Studies are highlighted in bold when they clearly attempted to account for hearing loss and italics when focused on a normal hearing group. Melcher et al. (2013) included participants that had clinically normal hearing but accounted for supra‐clinical frequencies (>8 kHz). Note that only animal studies with a confirmed behavioural measurement of tinnitus are included here. Where salicylate was used as the tinnitus inducer, this has been specified.

Subject type	Measure examined	Direction related to tinnitus	Studies
**Human**	Hippocampal/PHG volume	Increased	**Profant et al. (** **2020** **)**; **Elmer et al. (** **2023** **)**
		No change	**Husain et al. (** **2011** **)**; ***Melcher et al.* (** **2013** **)**; **Allan et al. (** **2016** **)**
		Decreased	*Landgrebe et al.* (2009); **Boyen et al. (** **2013** **)**; **Schmidt et al. (** **2018** **)**; Besteher et al. (2019)
	Parahippocampal microstructural abnormalities (ADC)	Increased	Gunbey et al. (2017)
	Hippocampal fractional anisotropy	Decreased	**Yoo et al. (** **2016a** **)**; **Gunbey et al. (** **2017** **)**
	Hippocampal gene enrichment	Increased	Bhatt et al. (2022)
	Hippocampal/PHG blood perfusion	Increased	*Laureano et al.* (2014)
		Decreased	Shulman et al. (1995); Shulman et al. (2007)
	Hippocampal/PHG glucose metabolism (PET)	Increased, related to tinnitus distress	**Schecklmann et al. (** **2013** **)**
		Increased, related to orofacially mediated tinnitus loudness	Lockwood et al. (1998)
	PHG connectivity with auditory cortex	Increased	*Maudoux et al.* (2012a); Maudoux et al., 2012b; **Schmidt et al. (** **2013** **)**; *Chen et al.* (2017)
	Hippocampal hubness	Increased	*Lan et al.* (2022)
	Scalp EEG PHG activity/connectivity	Increased	Vanneste, Heyning, and Ridder (2011); Vanneste, Plazier, et al. (2011); Song et al. (2015); **Vanneste and De Ridder (** **2016** **)**; De Ridder et al. (2023)
	Intracranial EEG hippocampal/PHG activity and connectivity	Decreased following residual inhibition (compared to baseline)	Sedley et al. (2015)
**Animal**	Hippocampal neurogenesis	Decreased after noise exposure, regardless of tinnitus status	Kraus et al. (2010)
		Decreased following salicylate	Niu et al. (2018)
	Dentate gyrus/CA1/CA3 acetylcholine	Decreased	Zhang et al. (2019)
	Dentate gyrus/CA1/CA3 vesicular GABA transporter	Decreased	Zhang et al. (2021)
	Hippocampal connectivity with auditory cortex	Increased following salicylate	Chen et al. (2015)
	CA1 spontaneous firing rates	Increased following salicylate	Ding et al. (2018)
	Hippocampal c‐Fos expression	Decreased following salicylate	Niu et al. (2018)

### 2.1 Structural changes in hippocampus and PHG in humans with tinnitus

Methods to measure cortical thickness/surface area and brain volume noninvasively with magnetic resonance imaging (MRI) have been popular in the characterisation of chronic disorders (e.g., major depression and bipolar disorder: Wise et al., 2017; pain: Liao et al., 2018). Because tinnitus is also a chronic condition these approaches have been applied to reveal related structural changes (Adjamian et al., 2014). In hippocampus, such changes include an increase in grey matter in tinnitus (Profant et al., 2020, or for opposite effects see Landgrebe et al., 2009). The study by Landgrebe et al. (2009) only included young tinnitus and control participants (<42 years) with hearing thresholds up to 25 dB HL from 125 Hz to 8 kHz, and so may only apply to this relatively uncommon tinnitus sub‐group, based on prevalence data (Al‐Swiahb & Park, 2016). Profant et al. (2020) found an increase in hippocampus and amygdala volume specifically correlated with tinnitus that was independent of age and hearing loss in this study sample. Notably, the effects of age and hearing loss play an important role, although even here the results are mixed across studies that attempt to account for these effects, with failures to replicate between and within laboratories (Allan et al., 2016; Boyen et al., 2013; Husain et al., 2011; Melcher et al., 2013; Profant et al., 2020; Yoo et al., 2016a). In PHG, effects have been observed related to tinnitus severity. In one study that controlled for age and hearing across different groups, left PHG cortical thickness decreased as a function of increased tinnitus severity (Schmidt et al., 2018).

The severity of tinnitus may be related to the common occurrence of psychiatric symptom comorbidity in the tinnitus population (Zoger et al., 2006). A recent study attempted to control for this psychiatric comorbidity and found that PHG grey matter volume was reduced in a low‐distress tinnitus group compared to an age‐matched no‐tinnitus control group (Besteher et al., 2019); however, the control group was not matched for hearing loss. Importantly, this study included an additional no‐tinnitus control group with psychiatric symptoms that were similar to the general comorbid symptom presentation in the tinnitus group. When both control groups were compared to the high‐ and low‐distress tinnitus groups a reduction in grey matter volume remained in the PHG, which was better predicted by tinnitus presence than psychiatric symptom scores. Further to this, another recent volumetric MRI study found that the head and body of the left hippocampus (as well as the left amygdala) in people with tinnitus were significantly larger than in control subjects when the groups had well‐matched hearing loss (Elmer et al., 2023). A large‐scale genome‐wide association study of UK Biobank data that included 38,525 subjects with tinnitus implicated gene upregulation (i.e., enrichment) in the hippocampus as being a factor in its presence (Bhatt et al., 2022).

Changes in the PHG have also been found in other neuroanatomical studies of both grey and white matter. Using diffusion tensor imaging data, Gunbey et al. (2017) found increased apparent diffusion coefficient values—measures of the movement of water through tissue, used as a proxy for microstructural abnormalities—in both parahippocampal regions in tinnitus patients (a mix of those with and without hearing loss) compared to normal hearing controls. This finding was correlated with tinnitus loudness, as assessed via a visual analog scale. In the same study, decreases in hippocampal fractional anisotropy values (measures of white matter microstructure) were evident in tinnitus patients compared to healthy controls, an effect which was greater in those with hearing loss compared to those without. Yoo et al. (2016a, 2016b) also studied fractional anisotropy values and found that they were negatively correlated with subjective tinnitus loudness in the right parahippocampus, but only in young tinnitus patients without any hearing loss.

The variable results in hippocampal structural changes associated with tinnitus may be a consequence of relatively low numbers of subjects in each comparison group, often between 10 and 15 and associated heterogeneity (Allan et al., 2016), or results may be influenced by the different analysis methods employed (Storelli et al., 2018). In summary, no consistent findings have been observed from measures of MTL brain structure in association with tinnitus, which may—at least in part—reflect heterogeneity, such as differing hearing levels, of the groups included in different studies.

In addition to correlational studies of brain structure or function with tinnitus characteristics, lesion studies can shed light on the role of damaged or resected areas by revealing the symptomatic consequences of that damage. A population in which acute and lasting changes in MTL can occur is patients who undergo surgical resection for treatment of medically refractory epilepsy. In one such case, resection of the epileptogenic focus (which included an area of the MTL covering only posterior portions of hippocampal and parahippocampal cortex) was followed by permanent cessation of the tinnitus percept contralateral to the resection (unpublished results from Sedley et al., 2015 data). However, the particular extent of surgery and location may be an important determinant of this outcome—an investigation of tinnitus prevalence in patients that experienced temporal lobe resection surgery for epilepsy treatment (which would to variable extents include portions of MTL including amygdala, hippocampus, and parahippocampus) found a doubling of rates of tinnitus compared to a healthy control population and a non‐resection epilepsy population (Paquette et al., 2017), though information on the specific extent of the resected tissue was not included. The authors inferred “that the MTL resection encroaching on the amygdala disrupts a noise‐cancellation system in the central nervous system” (Paquette et al., 2017), indicating that they were hypothesizing resection of the amygdala was a possible reason for this apparent doubling.

### 2.2 Functional changes in hippocampus and PHG in humans with tinnitus

Our understanding of brain function from the perspective of neuroimaging highlights how a system of multiple networks interacts to carry out functions, in both normal and disordered states. Some of the earliest brain imaging evidence in tinnitus came from case reports by Shulman and colleagues who used technetium‐99 m‐labelled single‐photon emission computed tomography (SPECT) imaging in their tinnitus clinic participants (Shulman et al., 1995). Shulman et al. (1995) summarised these findings as regional abnormalities in cerebral perfusion expressed as asymmetries and hypoperfusion across many regions of the brain, and highlighted the differences in MTL (amygdala, hippocampus, PHG). In a subsequent study, the pattern of MTL hypoperfusion/asymmetry was observed in 16/18 cases (Shulman et al., 2007). In a more recent report SPECT imaging in a group of moderately bothered tinnitus subjects with normal hearing (defined as <=25 dB in both ears, at and below 8 kHz) revealed increased perfusion of left PHG compared to a matched control group (Laureano et al., 2014).

SPECT imaging reveals brain activity at rest, through imaging blood perfusion, as opposed to glucose metabolism in fluorodeoxyglucose (FDG) positron emission tomography (PET), and a currently unknown combination of cerebral metabolic rate of oxygen, cerebral blood flow, and cerebral blood volume in BOLD fMRI. Nevertheless, resting‐state measures of brain activity have face validity in the case of tinnitus due to the chronic nature of tinnitus perception. BOLD fMRI does not involve radiation and provides higher resolution than the other neuroimaging techniques, and so has become more commonly adopted as a method of experimental investigation. This has led to the study of resting‐state networks, first using PET (e.g., Arnold et al., 1996; Fox & Raichle, 1984; Fox & Raichle, 1986), and later considered as temporally coincident patterns of connectivity across brain regions in fMRI (Biswal et al., 1995).

Ninety‐one individuals with chronic tinnitus underwent an FDG PET study and had clinical characteristics of duration and distress correlated to neuronal activation patterns (Schecklmann et al., 2013). Tinnitus duration correlated positively with right inferior frontal, right ventromedial prefrontal, and right posterior cingulate cortex. Parahippocampal and hippocampal areas and posterior inferior temporal gyrus were correlated to tinnitus distress, rated by tinnitus questionnaire score. Another PET study with O^15^‐water as the tracer examined four tinnitus patients able to alter the perceived loudness of the tinnitus by orofacial movements. That study revealed foci of increased activity in hippocampus, thalamus, and left middle and transverse temporal gyri (Lockwood et al., 1998), mediated according to orofacial movement‐related loudness changes in tinnitus. The authors noted that in subjects with tinnitus compared to controls, an increased sound‐evoked regional cerebral blood flow (rCBF) in the left primary auditory cortex, as well as an increased sound‐evoked rCBF in the left hippocampus, was observed. Although these results suggest abnormal auditory processing in tinnitus subjects, some differences might have been related to differences in age and hearing levels between the subject groups. The subjects with tinnitus had high‐frequency hearing losses varying from 30 to 70 dB while the control group had normal hearing levels. Nonetheless, this study demonstrates that a within‐subject design to examine changes in brain activity, wherein the tinnitus percept is modulated within an individual, allows for unique comparisons to be made. Furthermore, a recent resting‐state FDG‐PET study examining the consequences of hearing aid intervention in tinnitus found increased metabolism in right hippocampus, among other regions, when compared to pretreatment scans (Simonetti et al., 2022).

fMRI can only measure relative changes in brain activity over time, rather than an absolute values as SPECT and PET can, and therefore lacks a single straightforward analysis method for resting‐state measures, necessitating more complex and varied analyses based on patterns of correlation between brain regions. In the normal resting state, there is evidence that the hippocampus is associated with both the default mode network (DMN) and the somatomotor network (in particular the anterior aspect of the hippocampus; Ezama et al., 2021; Seoane et al., 2022), while the parahippocampus is found to be part of the DMN (Seoane et al., 2022; Ward et al., 2014) and visual and dorsal attention networks (Seoane et al., 2022). Evidence exists for the involvement of these same structures in brain networks in tinnitus (Salviati et al., 2014). This includes greater PHG connectivity to the auditory network than in a no‐tinnitus control group (Maudoux et al., 2012a; Maudoux et al., 2012b), and greater tendency for left hippocampus, left amygdala and left temporal pole to act as information hubs in the brain in chronic tinnitus patients without hearing loss, compared to a hearing‐ and age‐matched control group (Lan et al., 2022). There are also mechanistic‐level descriptions of hippocampus and PHG connectivity in tinnitus. Chen et al. (2017) measured resting‐state fMRI BOLD in a middle‐aged normal hearing tinnitus population compared to a matched control group and found a robust consistent pattern of altered effective connectivity in the tinnitus group from left and right hippocampus to respective ipsilateral regions: middle temporal gyrus, postcentral gyrus, and middle occipital gyrus. An additional finding in the tinnitus group was increased effective connectivity from right hippocampus to left primary auditory cortex, which also correlated positively with tinnitus duration. The authors suggested that this correlation may be interpreted as evidence for a tinnitus memory signal being sent to auditory cortex that is consolidated in chronic tinnitus. Given the existing evidence, it is difficult to determine the precise directionality of hippocampal activity for storage of the memory percept of tinnitus. That is, one possibility is that the memory trace itself is stored within auditory cortex, and hippocampus facilitates the persistence of that trace. Alternatively, the memory of tinnitus may well be stored within the hippocampus and thus connectivity to auditory cortex represents ongoing retrieval of that memory. There is still debate about long‐term memory trace storage in general within sensory cortex and hippocampus and the reciprocal nature means it is likely much more complex than outlined here (Kumaran & McClelland, 2012). However, it is worth noting that even if this was clarified for normal long‐term memory processes, it would still not be clear whether tinnitus reflected similar “normal” processes.

EEG studies have also shown that parahippocampal activity is related to the perceived laterality of the tinnitus (Vanneste, Heyning, & Ridder, 2011; Vanneste, Plazier, et al., 2011), being increased predominantly contralaterally to the tinnitus ear, and showing a stronger laterality correlation than auditory cortex activity. Another EEG study found differential PHG activity in tinnitus depending on the level of hearing loss (Vanneste & De Ridder, 2016), with a tinnitus group with more severe hearing loss having greater resting‐state parahippocampal activity than the tinnitus group with lesser hearing loss. However, we are not aware that any similar comparisons have been made in groups without tinnitus, so it is uncertain whether what is revealed in these results is a specific interaction involving hearing loss and tinnitus, or a direct effect of hearing loss alone. Evidence from an intracranial recording in an epilepsy patient undergoing clinical monitoring for potential seizure resection found that transient reductions in tinnitus loudness induced following external sound presentation (residual inhibition) were accompanied by: (1) reduced activity within the hippocampus and PHG, and (2) reduced functional connectivity between hippocampus and multiple brain regions (Sedley et al., 2015). The PHG may also be affected differently depending on the age of tinnitus onset; an EEG study revealed greater activity in the parahippocampus in the mid‐beta band (18.5–21 Hz) in a late‐onset group of patients compared to an early‐onset group (Song et al., 2015). Moreover, a recent source‐localised resting‐state EEG study demonstrated an increase in bidirectional connectivity between PHG and auditory cortex compared to non‐tinnitus subjects (De Ridder et al., 2023), although neither hearing thresholds nor duration of tinnitus were examined in this study.

### 2.3 Evidence for changes in hippocampus following potential tinnitus induction in animals

Several studies have demonstrated changes in both structural and functional activity of the hippocampus following noise exposure and/or confirmation of tinnitus presence in animals (many of which are thoroughly reviewed in Kapolowicz & Thompson, 2019). Briefly, following noise exposure, changes have been observed in hippocampal c‐Fos expression (Wallhausser‐Franke et al., 2003, gerbils, although a larger effect was evident in amygdala), hippocampal place cells (Goble et al., 2009, rats), and hippocampal dentate neurogenesis (Kraus et al., 2010, rats), and activity‐related cytoskeletal protein expression (Kapolowicz & Thompson, 2016, also known as Arc, a protein implicated in memory consolidation; Plath et al., 2006), while Cheng et al. (2016) found that changes in the hippocampus following moderate noise exposure in mice (80 dB SPL, 2 h per day for 1–3 weeks) were greater than those observed in auditory cortex. More recently, Zhang et al. (2019) found that a histological marker of acetylcholine expression decreased in the hippocampus of guinea pigs, 2 weeks following noise exposure. In animals that did not develop behavioural evidence of tinnitus 12 weeks following noise exposure, this acetylcholine marker had largely normalised but remained diminished in those that did develop behavioural evidence of tinnitus, particularly in the dentate gyrus, as well as CA1 and CA3 subregions. A follow‐up study examining a marker for the vesicular GABA transporter (VGAT) found that 12 weeks after noise exposure, exposed animals with no tinnitus (i.e., resistant to tinnitus) had similar or increased levels of VGAT compared to controls, while animals with tinnitus showed diminished VGAT labelling in the same areas as the previous study (dentate gyrus, CA1, CA3; Zhang et al., 2021).

Following salicylate administration in rats—a common inducer of acute tinnitus in animals (Berger et al., 2013; Cazals, 2000; Stolzberg et al., 2011)—Chen et al. (2015) demonstrated an enhancement in cortical evoked potentials, concurrent with an enhancement of local field potentials in the lateral amygdala, in rats with behavioural evidence of tinnitus. This also resulted in an increase in functional connectivity (as measured with resting‐state fMRI) between the hippocampus and structures at various levels of the auditory system. Similarly, a more recent study demonstrated that salicylate increased the mean spontaneous firing rate of rat hippocampal CA1 neurons (Ding et al., 2018). In a separate experiment with unexposed animals, stimulation of hippocampal CA1 neurons also modulated the response of 79% of auditory cortical neurons. Chronic administration of sodium salicylate to rats affected neurogenesis in the dentate gyrus of the hippocampus, concurrent with behavioural evidence of tinnitus and impairments in spatial memory ability (Niu et al., 2018).

At present, it is not clear whether or to what extent these changes in hippocampal structure and function relate to emotional distress in animals, although efforts to study the influence of affective state on hippocampus have been proposed (Poirier et al., 2019). Indeed, nearly all tinnitus animal studies do not examine the presence of distress, which is difficult to quantify in animals, although a recent study has suggested increased distress in animals with tinnitus (Lauer et al., 2018). Nonetheless, given the current evidence, it is purely speculative to assume that any changes in hippocampal morphology or function in animals relates to a form of distress due to the potential presence of tinnitus, following noise exposure or salicylate administration. Contrastingly, it has been known for many decades that animals—including rodents—are capable of long‐term memory formation, mediated by the hippocampus (e.g. Vnek & Rothblat, 1996). Intriguingly, changes in the hippocampus following noise exposure do not cause deficits in spatial memory in animals with tinnitus (Zheng et al., 2011), although such an effect was observed in noise‐exposed mice that were not tested for the presence of tinnitus (Liu et al., 2018). These results further suggest that noise‐induced hippocampal changes, when present with confirmed tinnitus, may have a more specific functional consequence than general memory impairment. Further studies implementing an animal behavioural model of distress may help to elucidate whether hippocampal changes reflect any level of emotional distress in relation to the tinnitus percept in animals, as opposed to an increase in the strength of an auditory memory trace independent of any emotional response.

## 3 A MODEL FOR PERSISTENT TINNITUS

A model implicating the hippocampus and other MTL structures in tinnitus, with a particular focus on its role in auditory memory, was originally put forward by Shulman (1995). This model, described as a final common pathway for tinnitus, was based around SPECT results showing evidence for perfusion asymmetries and hypoperfusion in the MTL of tinnitus patients, relative to non‐tinnitus subjects. Its primary focus was suggesting that the MTL was responsible for maintaining a paradoxical auditory memory that was perceived as tinnitus, which could explain the heterogeneity of tinnitus perception across individuals. The term paradoxical in this context was used to imply that it was an undesired retention of the memory. Subsequently, this model was expanded to highlight the affective components involved in tinnitus (Shulman et al., 2009), while a similar model extended to pain and other brain networks was proposed by de Ridder et al. (2011). As mentioned above, however, in all these models, emotional processes were intertwined with memory processing, making it hard to dissociate the precise role of the MTL. For the current article, we have focused on the mnemonic role of the MTL (specifically, hippocampus/PHG), based on current evidence. Importantly, the MTL is not necessary for the rich experience of emotional processing (Berridge & Kringelbach, 2013; Damasio et al., 2013), an aspect which is particularly strikingly emphasised by a case study of a patient whose MTL and insula (among other structures) were destroyed bilaterally by Herpes simplex encephalitis (Damasio et al., 2013).

Within the context of the current review, we can begin to consider specifically *how* the hippocampus may contribute to the persistence of tinnitus, from a perspective of maintaining the memory for the percept. From the outset, acute tinnitus is likely initiated by a particular auditory process, which could involve numerous theories directly implicating the auditory system, such as increased central auditory gain or increased central noise in response to a reduction in peripheral output (e.g. Schaette & McAlpine, 2011; Schilling et al., 2021; Zeng, 2020). It is worth noting here that research has highlighted, however, that increased spontaneous firing cannot fully explain the presence of chronic tinnitus, as noise‐exposed animals without behavioural evidence of tinnitus show similar increases, at least at the level of the inferior colliculus (Coomber et al., 2014; Longenecker & Galazyuk, 2016; Ropp et al., 2014), while a human magnetoencephalography study controlling for hearing loss only found evidence for tinnitus‐related changes in the low‐frequency delta band activity during resting‐state recordings, as opposed to the high gamma band that would be suggestive of hyperactivation (Adjamian et al., 2012); even in this case, significantly increased delta activity was only seen in the tinnitus with hearing loss group compared to the no tinnitus without hearing loss group. The perception of the tinnitus signal may then become chronic through mechanisms whereby the hippocampus and other MTL structures facilitate maintenance of this percept at the level of the auditory system. Several lines of evidence point towards the directionality of this connectivity. In one study using Granger causality analysis of resting‐state fMRI data, directional connectivity from hippocampus to auditory cortex was increased in patients with tinnitus (Chen et al., 2017). This relationship correlated with tinnitus duration, and was proposed to be related to the chronification and strengthening of the tinnitus percept. Within this study, the interpretation of this result was that the hippocampus was relaying the memory of the phantom sound to the auditory cortex for consolidation of the acoustic representation, although as mentioned above, it is still unclear where the specific memory trace may actually be “stored” per se. In an aforementioned recent EEG study (De Ridder et al., 2023), increased connectivity between PHG and auditory cortex in the tinnitus group was found to be bidirectional, although it is unclear how the duration of tinnitus affected this result. A related finding in Schmidt et al. (2013) was that the mildly bothersome chronic tinnitus group showed increased connectivity between PHG and auditory cortex compared to the no‐tinnitus group. Under this model, the emotional response to this chronic percept is therefore a consequence of experiencing the memory of the tinnitus signal. However, the emotional response itself or a prior state of high stress may indeed enhance the tinnitus percept (Mazurek et al., 2012), creating a self‐perpetuating feedback loop.

There are two accounts of how the maintenance of tinnitus may be maintained by the MTL in the context of a predictive coding framework, both of which could co‐occur. In the case of de Ridder et al. (2014), the auditory cortex lacks ascending input, and therefore “searches for” the missing input from other sources, and draws this from auditory memory, a process which may be mediated via the hippocampus and PHG. This auditory memory then becomes the pervasive prediction, resulting in the persistence of chronic tinnitus. In the scenario proposed by Sedley et al. (2016), some degree of loss or peripheral input, along with other factors, causes an increase in the magnitude and precision of spontaneous activity. In a normal scenario, this does not present as a conscious auditory percept, but tinnitus occurs when higher generative models are adjusted to accept and predict this activity as a sound source, a factor potentially mediated by hippocampus/PHG. Persistence of these predictions may then lead to the persistence of tinnitus. Somewhat related to this, an intriguing study of a small group of tinnitus patients (*n* = 6) found that injection of amobarbital into an artery that supplies the amygdala and hippocampus could suppress pure tone tinnitus but not white noise tinnitus (De Ridder et al., 2006), suggesting that there may be differences in how the hippocampus contributes depending on the nature of the percept. Given that the hippocampus is implicated in replay of memories and in continual learning (Stoianov et al., 2022), as well as in auditory working memory (Kumar et al., 2021), one prospect of this is that tinnitus may effectively become a learned stimulus that is a continuously replayed memory of a sensory experience, which may be strengthened or weakened according to how well that particular percept can be encoded and replayed.

**FIGURE 2:**
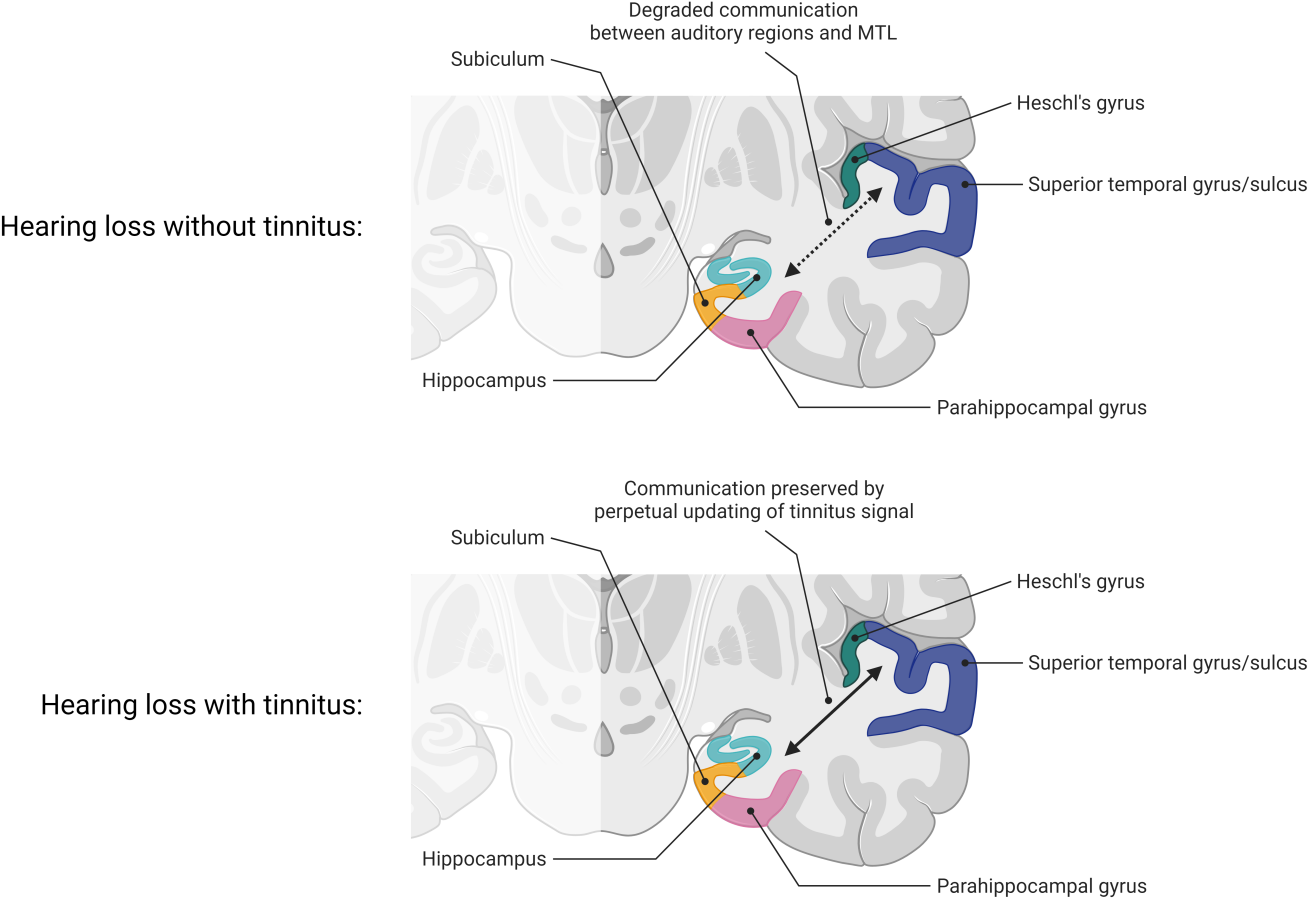
The proposed model by which hippocampal activity may be preserved when hearing loss is present with tinnitus. In one of the models (Model 2) put forward in Griffiths et al. (2020), some hippocampal dysfunction may occur as a result of diminished auditory input, which results in degraded communication between auditory cortex and MTL regions (top panel). In the scenario where tinnitus is maintained by an ongoing prediction of the memory of a phantom signal (bottom panel), communication between MTL regions is at least somewhat preserved, due to a perpetual updating of this signal in both directions; predictions held in memory update to correspond with ongoing auditory cortex activity, and sensory representations in auditory cortex are adjusted in light of predictions in memory.

One may then consider whether tinnitus can be present without prior memory for an external sound with the same characteristics. Indeed, in congenitally deaf animals and humans, tinnitus appears to be absent (Eggermont & Kral, 2016; Lee et al., 2017; Knipper et al., 2020). Contrastingly, people with missing limbs at birth (aplasia) can experience phantom limb syndrome (Melzack et al., 1997), which has previously been highlighted as having many parallels with tinnitus (Muhlnickel et al., 1998). To our knowledge, there is a lack of evidence involving the hippocampus or other MTL structures in phantom limb syndrome, despite the condition being exacerbated by the presence of emotional distress (Sherman et al., 1989) and the condition itself being related to increased emotional distress, albeit confounded with comorbid factors (Whyte & Niven, 2001), in a similar manner to that of tinnitus. This suggests some degree of important divergence between the two conditions in terms of pathological brain changes, and further supports the argument for a critical role of the hippocampus in chronic tinnitus perception, given the growing literature demonstrating changes following deafferentation and tinnitus presence. A recent study found an interesting and somewhat surprising result that, when present with hearing loss, tinnitus was associated with an improvement in cognitive performance (compared to hearing loss alone) in a non‐Hispanic elderly population (Hamza & Zeng, 2021), suggesting that the presence of tinnitus is somehow countering the known risk factor of hearing loss in cognitive decline (Lin et al., 2013) in some populations, although curiously a Hispanic population did not show this effect—the reasoning behind this demographic disparity was not explored within that study. Within the context of the current review, this may lead one to speculate whether hippocampal involvement in tinnitus plays a role, where the persistent auditory memory of the tinnitus signal preserves hippocampal function and prevents previously demonstrated diminished hippocampal neurogenesis in the presence of hearing loss. Placing this within one of four recently posited mechanisms relating dementia to hearing loss (Griffiths et al., 2020), this would fit within a mechanism whereby there is a function‐pathology interaction between the MTL and the auditory cortex (mechanism 2 in Griffiths et al). In normal hearing, there would be meaningful correspondence between auditory signals and predictions mediated by MTL, something which is diminished in the presence of degraded input, as in the case of hearing loss. Thus, one hypothesis would be that tinnitus effectively restores this correspondence or counteracts any diminished communication by requiring a perpetual updating of information between hippocampus and auditory cortex, thus effectively keeping the hippocampus functionally engaged (Figure 2). Further studies are required to elucidate the mechanisms behind this, but the impact could be profound if the results of Hamza and Zeng (2021) are indeed reproducible. One possible candidate mechanism is that the correspondence of representations in MTL and auditory cortex reduces ongoing prediction errors in these areas.

It is worthwhile to note here that we have focused here on a memory‐based role for the MTL in tinnitus perception. This does not necessarily conflict with the fact that tinnitus often results in an affective response, but suggests that the critical role of the MTL is mnemonic rather than affective processing. As in the case of familiar music, an emotional reaction to particular external sounds may be influenced by the prior memory of those sounds, mediated by the MTL (Samson & Peretz, 2005). In this scenario, tinnitus distress would relate to the strength of the memory percept (rather than the perceived level of the tinnitus), driven by the MTL, which would be associated with a heightened emotional response to this memory, likely mediated by frontal brain regions (Dias et al., 1996; Glotzbach et al., 2011; Tyng et al., 2017). In support of this idea, some of the studies presented above highlight a relationship between MTL structures and tinnitus‐related distress (e.g., Schecklmann et al., 2013).

While we have argued that the hippocampus may maintain the percept of a tinnitus trace, this does not preclude the structure from simultaneously performing other roles, potentially based on other mechanisms not discussed thus far. Parallel processing streams in the hippocampus can support multiple cognitive functions (Lee et al., 2020), not all of which depend on ongoing neuronal firing (Beukers et al., 2021). Additionally, the question arises as to whether the experience of tinnitus might be considered a type of memory as opposed to a type of ongoing perception. Both types of experience could be subserved by the same neural architecture, including high‐level sparse representations in the hippocampus that cause activity in sensory cortex usually associated with perception. That said, clearly further experimental research is required to determine specifically *how* the hippocampus may maintain the perception or memory of an ongoing phantom representation of an auditory object.

The current hypothesis relies on the premise that tinnitus is related to a persistent memory of an auditory object, one which is phantom in nature. If this idea was to be falsified, then the role of the hippocampus in maintaining the memory of a phantom auditory object (i.e., tinnitus) would then become questionable. Additionally, here we have intentionally focused particularly on the hippocampal system, to consider the nature of tinnitus as a persistent memory, a cognitive process in which the hippocampus/PHG are most‐commonly implicated, rather than discussing in detail other mechanisms behind tinnitus that are undoubtedly relevant. These mechanisms have implicated several other brain regions beyond the hippocampus and auditory system, including nucleus accumbens, ventromedial prefrontal cortex, thalamic reticular nucleus, middle frontal gyrus, fornix, and precuneus (Leaver et al., 2011; Rauschecker et al., 2010; Rauschecker et al., 2015; Rosemann & Rauschecker, 2022; Rosemann & Rauschecker, 2023a; Rosemann & Rauschecker, 2023b), cerebellum (Bauer et al., 2013; Mennink et al., 2022), insula (Chen et al., 2023; Lenhardt et al., 2008), and cingulate cortex (Chen, Liu, et al., 2018; Golm et al., 2013). These structures likely play roles in tinnitus that are not necessarily mutually exclusive, including—but not limited to—those involved in persistence of a memory trace, gating of pathological auditory activity and emotional responses to a chronic phantom percept.

## 4 SUMMARY

There is a growing wealth of evidence implicating substructures of the hippocampus and MTL in tinnitus, both from animals and from humans. The data as a whole suggest that these structures may underlie persistence of a memory trace for tinnitus. With further research and evidence accumulation, treatment strategies could be constructed to attempt to alter this long‐term memory, aiming to diminish or eliminate the underlying percept itself, rather than just the reaction to the percept. Further research should aim to determine whether pathological changes in MTL activity are indeed required for the persistence of tinnitus, and test the hypothesis that these structures play a major role in memory for an auditory object.

## CONFLICT OF INTEREST STATEMENT

J.I.B. has consulted for Turner Scientific on a behavioural model of tinnitus.

## Data Availability

Data sharing not applicable to this article as no datasets were generated or analysed during the current study.
